# GeneXpert MTB/RIF Ultra performance to detect uncommon *rpoB* mutations in *Mycobacterium tuberculosis*

**DOI:** 10.1186/s13104-023-06394-z

**Published:** 2023-07-14

**Authors:** Leen Rigouts, Jelle Keysers, Reenaers Rabab, Kristina Fissette, Armand van Deun, Bouke Catherine de Jong

**Affiliations:** 1 Institute of Tropical Medicine, Mycobacteriology Unit, Nationalestraat 155, Antwerp, Belgium; 2 Independent Consultant, Louvain, Belgium

**Keywords:** GeneXpert MTB/RIF Ultra, *rpoB*, Rifampicin resistance, Tuberculosis

## Abstract

**Objective:**

To investigate the performance of GeneXpert MTB/RIF Ultra to accurately detect rifampicin resistance for less common *rpoB* mutations that potentially confer phenotypic resistance, we tested 28 such *Mycobacterium tuberculosis* cultures with Xpert Ultra.

**Results:**

They represented 22 different (combinations of) *rpoB* mutations. Of 28 isolates tested, one was reported by Xpert Ultra as “No rifampicin resistance detected”, 8 yielded a “Rifampicin indeterminate” result, and 19 were identified as rifampicin resistant. Overall, our results corroborate previous observations on the “Indeterminate” results for mutations at codon 432, while we add Lys446Gln as additional “Indeterminate” result and Pro439Leu as a false rifampicin-susceptible result. Furthermore, we document other uncommon point mutations and indels across the *rpoB* gene that are mostly correctly identified as rifampicin resistant by Xpert ultra (V3). Taken together, “Indeterminate” results in Xpert Ultra may indicate underlying *rpoB* mutations within the rifampicin-resistance determining region and thus increase the post-test probability of rifampicin resistance, albeit to an unknown extent.

**Supplementary Information:**

The online version contains supplementary material available at 10.1186/s13104-023-06394-z.

## Introduction

The GeneXpert MTB/RIF assay (Cepheid, USA) has been endorsed by the World Health Organization as initial test for the detection of (rifampicin-resistant, RR) *Mycobacterium tuberculosis* (MTB) [1]. The latest version of this assay (GeneXpert MTB/RIF Ultra; hereafter Xpert Ultra), has increased specificity for RR calling through melting temperature (Tm) analysis, compared to its predecessor that was based on delayed or absent probe binding [2]. Xpert Ultra uses a combination of four sloppy molecular beacon probes, resulting in reproducible and measurable Tm shifts in presence of RR-causing *rpoB* mutations [2]. Nevertheless, some systematic errors have been reported for Xpert Ultra as well, both false-RR results due to presence of synonymous mutations, and false rifampicin-susceptible (RS) results for mutations in the codons 431–433 region, such as Gln432Leu and Gln432Pro [3]. In addition, the in silico Tm analysis performed as part of the Xpert algorithm does not always correctly predict the specific mutation that confers RR [4].

To further investigate the performance of Xpert Ultra for less common mutations, we searched our internal database with Sanger sequence data for infrequent *rpoB* mutations in the Ultra target region, focusing on those that had shown to yield false-RS Xpert results in other studies, and those for which no Xpert Ultra data has been published yet.

## Isolates and methods

At the Mycobacteriology Unit of the Institute of Tropical Medicine (Antwerp, Belgium), we have applied *rpoB* Sanger sequence analysis on clinical specimens and MTB isolates, since around 2004, both for patient management purposes as for research and clinical trials. The dataset investigated in this study comprised of 3672 records from about 3500 different patients originating mostly from Asian and African countries in the period of 2014 to 2021. A search for any Sanger-detectable mutation from codons 424 to 454, resulted in 94 different mutation types in 1429 records, many of them detected directly in the clinical specimen. Narrowing down the selection criteria, we identified 29 (2%) records with previously documented false-RS Xpert Ultra results. Finally, from the selected mutants, 28 MTB isolates could be retrieved from our − 80 °C collection, representing 22 different (combinations of) *rpoB* mutations (Table 1 and Additional file 1). The individual variants included 3 different non-frameshift insertions or deletions (indels), and 27 non-synonymous single nucleotide polymorphisms (SNPs). Nine isolates had a combination of 2 SNPs (combo), of which 2 combined one SNP within and one outside the Ultra target region. The remaining 16 isolates caried a single SNP. These 16 singletons represented 10 different SNPs, of which 3 were classified as “Associated with resistance” (Ass-w-R) according to the WHO mutation catalogue (Version 1) and 7 as “Associated with resistance-interim” (Ass-w-R-int) [5]. Among the isolates with “combo” SNPs, 7 combined an Ass-w-R mutation with an Ass-w-R-int (n = 5) or a mutation with “uncertain significance” (n = 2), while the two remaining combined different Ass-w-R-int mutations or no data was available in the catalogue.

**Table 1:** GeneXpert Ultra, phenotypic drug-susceptibility and *rpoB* sequencing results for 28 selected *Mycobacterium tuberculosis* isolates

Culture number	rpoB mutation(s)	Type of mutation	Appearance	WHO catalogue V1		pDST		GeneXpert Ultra (V3) result	
				Final confidence grading	Presence among (SOLO)RR isolates	LJ	MGIT	Report	Per probe analysis based on Tm for combo mutants
CT2005-00009	Ins431(agCCAc); P439L	INDEL-no fs	Combo	No data; Ass w R-int	No data; 0	RMP-R	NT	No R detected	No R detected; No R detected
CT2008-02955	Del427-428(ACCAGC)^a^	INDEL-no fs	Single	Not ass w R^b^	No data	RMP-S^e^	RMP-S^d^	Indeterminate	
CT2005-02316	Del437(AAC)	INDEL-no fs	Single	Ass w R-int^b^	No data	RMP-R	RMP-R	R detected	
CT1993-09583	H445R; N487H^a^	SNP	Combo	Ass w R; uncert sign	79; 2	RMP-R	RMP-R	R detected	R detected; not applicable
CT2007-00288	D435Y; P454L	SNP	Combo	Ass w R; uncert sign	44; 6	RMP-R	RMP-S^d^	R detected	R detected; R detected?
CT1998-00166	S428R; H445R	SNP	Combo	Ass w R-int; Ass w R	5; 79	RMP-R		R detected	R detected; R detected
CT2014-02522	Q429L; D435V	SNP	Combo	Ass w R-int; Ass w R	9; 732	RMP-R		R detected	R detected; R detected
CT2004-01679	S431G; S450W	SNP	Combo	Ass w R-int; Ass w R	6; 151	RMP-R		R detected	R detected; R detected
CT1997-01244	M434I; D435Y	SNP	Combo	Ass w R-int; Ass w R	16; 162	RMP-R		R detected	R detected; R detected
CT2014-00803	N437S; R447P	SNP	Combo	Ass w R-int; no data	2; no data	RMP-R		R detected	R detected; R detected
CT2009-01036	M434T; H445D	SNP	Combo	Ass w R-int^b^; Ass w R	No data; 268			Indeterminate	Indeterminate; R detected
CT2014-00781	D435N; S450V	SNP	Combo	Ass w R-int^b^; Ass w R-int	No data; 2	RMP-R		R detected	R detected; R detected
CT2012-00752	Q432L	SNP	Single	Ass w R	21	RMP-R		Indeterminate	
CT2014-02541	Q432L	SNP	Single	Ass w R	21	RMP-R		Indeterminate	
CT2008-01914	Q432K	SNP	Single	Ass w R	34		RMP-R	R detected	
CT2014-01261	Q432K	SNP	Single	Ass w R	34	RMP-R		R detected	
CT2013-01926	Q432P	SNP	Single	Ass w R	29	RMP-R	NT	Indeterminate	
CT2015-00488	Q432P	SNP	Single	Ass w R	29	RMP-R		Indeterminate	
CT2018-03321	S428R	SNP	Single	Ass w R-int	5	RMP-S^c^	RMP-S^d^	R detected	
CT2019-04011	Q432E	SNP	Single	Ass w R-int	1	RMP-R		R detected	
CT2014-00323	D435A	SNP	Single	Ass w R-int	2	RMP-S^c^	RMP-S^d^	R detected	
CT2015-00705	D435A	SNP	Single	Ass w R-int	2	RMP-S^c^	RMP-S^d^	R detected	
CT1997-01460	H445P	SNP	Single	Ass w R-int	5	RMP-R	RMP-R	R detected	
CT2008-01398	H445P	SNP	Single	Ass w R-int	5	RMP-R		R detected	
CT2014-00787	K446Q	SNP	Single	Ass w R-int	7	RMP-R		Indeterminate	
CT2014-00789	K446Q	SNP	Single	Ass w R-int	7	RMP-R		Indeterminate	
CT2014-00761	N438K	SNP	Single	Ass w R-int^b^	No data	RMP-R		R detected	
CT2013-00428	G442R	SNP	Single	Ass w R-int^b^	No data	RMP-R		R detected	

*Ass-w-R(-int)* associated with resistance (interim), *indel* insertion/deletion, *no fs* no frameshift, *pDST*  phenotypic drug-susceptibility testing, *LJ* Löwenstein-Jensen medium (40 µg/ml), *MGIT*  Mycobacteria Growth Indicator Tube (1 µg/ml or 0,5 µg/ml for^4^), *SNP* Single nucleotide polymorphism, *RMP* rifampicin, *R* resistant(ce), *S* susceptible

^a^Outside XpertUltra target region

^b^No data in WHO catalogue, but applying WHO rule on own pDST results

^c^MIC ≤ 10 µg/ml

^e^MIC of 20 µg/ml

MTB isolates were retrieved from the freezer, and analyzed by Xpert Ultra (V3) by transferring some material from the frozen stock directly in the sample reagent. One third of the isolates did not yet have phenotypic drug-susceptibility data, and were tested in the MGIT960 system (0.5 µg/ml; Becton Dickinson, USA) and on Löwenstein-Jensen (LJ; two-fold serial dilutions from 10 to 160 µg/ml), after regrowing from the frozen stock on LJ medium. For the others, the initial, routinely obtained phenotypic result was considered (LJ 40 µg/ml or MGIT 1.0 µg/ml).

## Results and discussion

Of 28 isolates tested, one was reported by Xpert Ultra as “No R detected”, eight yielded an “R Indeterminate” result, and 19 were identified as RR (Table 1).

The reported RS isolate had a combo of Ins431(agCCAc) and Pro439Leu (ccg > ctg), and yielded somewhat deviating Tms for both probes 1 and 2 (68.3 and 72.9 °C respectively), but still resulted in a wild type interpretation for both probes (See Additional file 1). Pro439Leu is classified as Ass-w-R-int, while the Ins431(agCCAc) insertion is not listed in the WHO catalogue. Since the isolate was phenotypically resistant on LJ medium at initial and repeated, contemporaneously testing, this case is considered a false-RS result by Xpert Ultra.

From the 8 isolates with an “R Indeterminate” result, six were singleton SNPs that each occurred twice: Gln432Leu, Gln432Pro and Lys446Gln. For codon-432 mutants no Tm was obtained for probe 1 despite successful amplification, while for Lys446Gln the amplification of probe 3 failed (See Additional file 1). The 432 mutants are classified as Ass-w-R, while Lys446Gln is Ass-w-R-Int. All six were RR on LJ medium. Nevertheless, Xpert Ultra failed to identify them as RR, which confirms previous observations of not detecting some codon-432 mutants [3]. The Gln432Lys and Gln432Glu mutations were correctly identified as RR by Ultra.

Also for the combo Met434Thr/His445Asp an “R Indeterminate” result was reported, despite the fact that it correctly identified His445Asp as resistant (Tm of 71.9 °C for probe 3; See Additional file 1). While we would have expected an impact from Met434Thr on the Tm of probe 1, this was not the case. Rather, probe 2 did not have a successful Tm despite successful amplification (Ct 17.8). This is in line with observations from Ng and colleagues, where codon-434 mutations only impacted probe 2 and not probe 1 [6]. However, in our case no Tm was generated. This isolate was resistant on LJ and MGIT. Xpert Ultra failed to identify this combo as RR.

As for the last “R Indeterminate” case, the no-frameshift deletion at the edge of the targeted region (Del427-428(ACCAGC) prohibited amplification of probe 1, while the other probes had cycle threshold (Ct) values between 17.1 and 20.3 (See Additional file 1). Remarkably, this isolate was found phenotypically RS in MGIT (0.5 µg/ml) and on LJ with a minimal inhibitory concentration (MIC)_99_ of 20 µg/ml and MIC_100_ of 40 µg/ml. Hence, it seems that this deletion does not cause RR, and at most could be classified as “uncertain significance” or even “Not associated with resistance”. Hence, the obtained Xpert Ultra “R Indeterminate” seems acceptable until more certainty around the relevance of this insertion arises.

Among the 19 *rpoB* variants reported as RR by Xpert Ultra, three isolates appeared phenotypically RS by both LJ (MIC ≤ 10 µg/ml on LJ medium) and MGIT (0.5 µg/ml) testing: one with Ser428Arg and two with Asp435Ala. The Ultra-RR result was based on the predicted Tm from probe 1 or probe 2 respectively. In the WHO mutation catalogue, Ser428Arg has been observed in 5 RR isolates, while not being present among 24,433 RS isolates. Overall, it is known that elusive *rpoB* mutations can be miss-classified as RS by rapid or imprecise pDST [7]. Few pDST data for Ser428Arg mutants are publicly available though. El Maraachi and colleagues reported a MIC of 5 µg/ml in MGIT [8], justifying its classification as Ass-w-R-Int. More data would be needed to investigate the impact of these mutations.

Overall, our results corroborate the observations of Omar and colleagues regarding the “Indeterminate” results for SNPs at codon 432, while we add Lys446Gln as additional “Indeterminate” result and Pro439Leu as a false-RS result. Furthermore, we document other uncommon SNPs and indels across the *rpoB* gene that are mostly correctly identified as RR by Xpert ultra (V3). Missing RR at diagnosis may delay timely initiation of appropriate treatment with the risk of worse treatment outcome and continued RR-TB transmission, and from a public health perspective even diagnostics-driven selective advantage for less common mutations, as was probably the case for the *rpoB*_Ile491Phe mutation [9].

On the other hand, we identified two Ass-w-R-Int *rpoB* SNPs (Asp435Ala, Ser428Arg) that do not seem to cause phenotypic RR on their own. If these mutations are truly phenotypic/clinical RS, the Xpert Ultra generates a false-RR result, with potential unnecessary initiation of multi-drug resistant treatment. The associated risk for the patient may be limited if a safe, short multi-drug resistant TB regimen can be offered.

It must be noted that most of these mutations are uncommon. Their reported frequency as singletons in the WHO mutation catalogue varied from not being reported (n = 9 different mutations within the Ultra target region), to being reported between two and 706 times, compared to 6256 occurrences for the most common Ser450Leu mutation [5]. In our convenient study population, they represented 2% of the RRDR variants. Hence the impact of missed or non-reported RR with delayed treatment adaptation on one hand, or overtreatment with second-line drugs for erroneous RR calling for the investigated mutations will be limited on the global scale. Nevertheless geographical variations in mutation prevalence may occur, and may lead to discordant results for rifampicin-susceptibility testing, complicating patient management. Despite these minor shortcomings, we still fully support the use of rapid molecular testing for detection of RR-TB, acknowledging that none of the tests is perfect.

## Limitations

The number and variability of *rpoB* mutants tested in our study is limited because we had a focus on uncommon mutants based on our own Sanger sequence data set and only included MTB mutants that could be retrieved from our − 80 °C collection. Hence, this is not a representative sample for a clinical setting. It aims to extend the knowledge base on Xpert Ultra performance for these uncommon mutations.

## Supplementary Information

**Additional file1: Table S1.** Gene expert ultra phenotypic drug-susceptibility and *rpoB* sequencing results for 28 selected *mycobacterium* *tuberculosis* isolates.

## Data Availability

Upon reasonable request raw data can be made available.

## Abbreviations

Ass-w-R: Associated with resistance
Ass-w-R-Int: Associated with resistance interim
Ct: Cycle threshold
Indels: Insertions or deletions
ITM: Institute of Tropical Medicine, Antwerp, Belgium
LJ: Löwenstein-Jensen medium
MTB: *Mycobacterium tuberculosis*
TB: Tuberculosis
RS: Rifampicin-susceptible
RR: Rifampicin-resistant
SNP: Single nucleotide polymorphisms
Tm: Melting temperature
Uncert: Uncertain significance
WHO: World Health Organisation
Xpert (Ultra): GeneXpert MTB/RIF (Ultra) (Cepheid, USA)
